# VESTA: A SMARTPHONE-BASED ASSESSMENT OF MODERN FINANCIAL CAPACITY FOR OLDER ADULTS

**DOI:** 10.1093/geroni/igad104.2210

**Published:** 2023-12-21

**Authors:** Samuel Van Vleet, Rebekah Griffin, William Mallender, Matthew Fontanese, Haru Sakai, Blake Savage, Allyson Coldiron, Michael Barnett

**Affiliations:** Miami University, Oxford, Ohio, United States; University of Texas at Tyler, Tyler, Texas, United States; University of Texas at Tyler, Tyler, Texas, United States; University of Texas at Tyler, Tyler, Texas, United States; The University of Texas at Tyler, Tyler, Texas, United States; University of Texas at Tyler, Tyler, Texas, United States; University of Texas at Tyler, Tyler, Texas, United States; University of Texas at Tyler, Tyler, Texas, United States

## Abstract

Older adults are often targeted by financial scammers who exploit people unfamiliar with financial technologies and who may struggle to identifying fraudulent activity. Financial technologies have undergone significant changes in recent decades, yet financial capacity measures often do not reflect this shift. There is a clear need for innovating the measurement of financial capacity, particularly among older adults. VESTA (Virtual Economic Simulation and Trust Assessment) is a novel smartphone application that measures older adults’ susceptibility to different forms of financial exploitation. VESTA plays out like a smartphone game in which the user furnishes a virtual household. As the user conducts these virtual transactions, they are tasked with spending within a budget, monitoring bank charges, disclosing personal information securely, and determining whether incoming messages (i.e., texts, emails, and voicemail messages) are fraudulent or legitimate. The fraudulent charges and messages include contextual clues that mirror real-life scams. VESTA was designed to assess the user’s ability to manage money, identify incorrect information, and judge who to trust independent of memory ability. VESTA therefore purposefully targets severely overlooked pitfalls that impact older adults’ ability to identify fraudulent activity. Furthermore, VESTA utilizes a user-friendly, accessible design with large font and complete navigation with the simple press of a finger. As the migration of financial management and communication continues towards predominantly digital landscapes, this research and diagnostic tool represents an evolution of existing measures of financial capacity for an at-risk aging population.

